# The Non-Proliferative Nature of Ascidian Folliculogenesis as a Model of Highly Ordered Cellular Topology Distinct from Proliferative Epithelia

**DOI:** 10.1371/journal.pone.0126341

**Published:** 2015-05-22

**Authors:** Karim Azzag, Yoann Chelin, François Rousset, Emilie Le Goff, Camille Martinand-Mari, Anne-Marie Martinez, Bernard Maurin, Martine Daujat-Chavanieu, Nelly Godefroy, Julien Averseng, Paul Mangeat, Stephen Baghdiguian

**Affiliations:** 1 Université de Montpellier, Place Eugène Bataillon, 34095, Montpellier, Cedex 5, France; 2 Institut des Sciences de l’Evolution (ISE-M), CNRS, Montpellier, France; 3 Laboratoire de Mécanique et Génie Civil (LMGC), CNRS, Montpellier, France; 4 Institut de Génétique Humaine (IGH), CNRS, Montpellier, France; 5 INSERM U1040, Montpellier, France; 6 CHU Montpellier, Institut de Biothérapie, Montpellier, France; 7 Centre de Recherche de Biochimie Macromoléculaire (CRBM), CNRS, Montpellier, France; Laboratoire Arago, FRANCE

## Abstract

Previous studies have addressed why and how mono‐stratified epithelia adopt a polygonal topology. One major additional, and yet unanswered question is how the frequency of different cell shapes is achieved and whether the same distribution applies between non-proliferative and proliferative epithelia. We compared different proliferative and non-proliferative epithelia from a range of organisms as well as *Drosophila melanogaster* mutants, deficient for apoptosis or hyperproliferative. We show that the distribution of cell shapes in non‐proliferative epithelia (follicular cells of five species of tunicates) is distinctly, and more stringently organized than proliferative ones (cultured epithelial cells and *Drosophila melanogaster* imaginal discs). The discrepancy is not supported by geometrical constraints (spherical versus flat monolayers), number of cells, or apoptosis events. We have developed a theoretical model of epithelial morphogenesis, based on the physics of divided media, that takes into account biological parameters such as cell‐cell contact adhesions and tensions, cell and tissue growth, and which reproduces the effects of proliferation by increasing the topological heterogeneity observed experimentally. We therefore present a model for the morphogenesis of epithelia where, in a proliferative context, an extended distribution of cell shapes (range of 4 to 10 neighbors per cell) contrasts with the narrower range of 5-7 neighbors per cell that characterizes non proliferative epithelia.

## Introduction

The polygonal structure of cell layer has exerted a unique fascination among biologists since the original observations of Robert Hooke in 1665 [1]. The polygonal shape of epithelial cells represents one of the most remarkable landmarks of morphogenesis found in animals and plants [2]. Epithelial morphogenesis is the result of cross-talks between genetic determinism [3], the subsequent triggered molecular events [3] and physical topological constraints [4,5]. The polygonal topology directly impacts fundamental cellular processes such as apoptosis [6], coordinated migration [7], or orientation of cell division axis [8,9]. In the latter study the authors have designed a quantification method, which is based on the frequency distribution of cellular polygons to describe the topological characteristics of proliferative epithelia.

However, a general principle to account for the regularity of the cellular organization in different tissues, individuals and species is incomplete. Specifically, all previous studies dealt with proliferative epithelia and until now no data were available to illustrate how tissues can be organized without any input of mitotic events. We previously became interested as to how the follicular cells that covered ascidians eggs were subjected to apoptosis following fertilization [10]. In *Ciona intestinalis*, the follicular cells are organized as an external layer of cells that adhere to an extracellular matrix, the chorion, to which a second layer of cells (test cells) is associated with at the inner face. We described how one large follicular cell extends on the surface of the extracellular matrix respectively faced by 20 much smaller test cells, and how, 16h post-fertilization, apoptosis of the 20 test cells was topologically controlled by each facing unique follicular cell [11]. We also described that the symmetrical organization of *Ciona intestinalis* follicular cell system respects physical rules, that could be simply simulated by multiple symmetries organizing 60 cells (the number of follicular cells in *Ciona intestinalis*) covering a sphere through lateral cell-cell contacts with 5 (pentagons) and 6 (hexagons) neighbors. Such an idealistic distribution appeared clearly different than from characterized previously for *Drosophila melanogaster* wing disc [12], and which was later extended to proliferative epithelia from cucumber to mammals [5]. Here we have characterized further and quantified the topological organization of the follicular cell layer from five ascidian species with the aim to gain answers to the following questions. What is the origin of ascidian folliculogenesis? What are the quantitative characteristics of the topological organization of *Ciona intestinalis* follicular cells and of other ascidians species? Do the quantitative data converge or diverge to the frequency distribution observed in known models of proliferative epithelia? Is it possible to simulate the data with simple physics laws? The different answers to these questions are: first, folliculogenesis resulted from a non-proliferative and non-apoptotic accretion mechanism taking place in the gonads; second, the frequency distribution of cell shapes is based on a majority of hexagons, then pentagons and a few heptagons; third, this characteristic frequency is shared by and conserved in other species of ascidians and is independent of the total number of follicular cells covering the spherical oocyte and/or the extent of surface covered by a single cell; fourth, the frequency distribution of cell shapes in these ascidian models of non-proliferative epithelia is significantly different of models of planar or spherical proliferative epithelia that were invalidated or not for apoptosis; fifth, computer simulations that mimicked the successive steps of epithelial morphogenesis in either a proliferative or non-proliferative context were developed, and the results of these simulations confirmed that a few physical principles govern the distribution frequency of cell shapes for both proliferative contexts.

## Materials and Methods

### Egg collection

**Fig 1:**
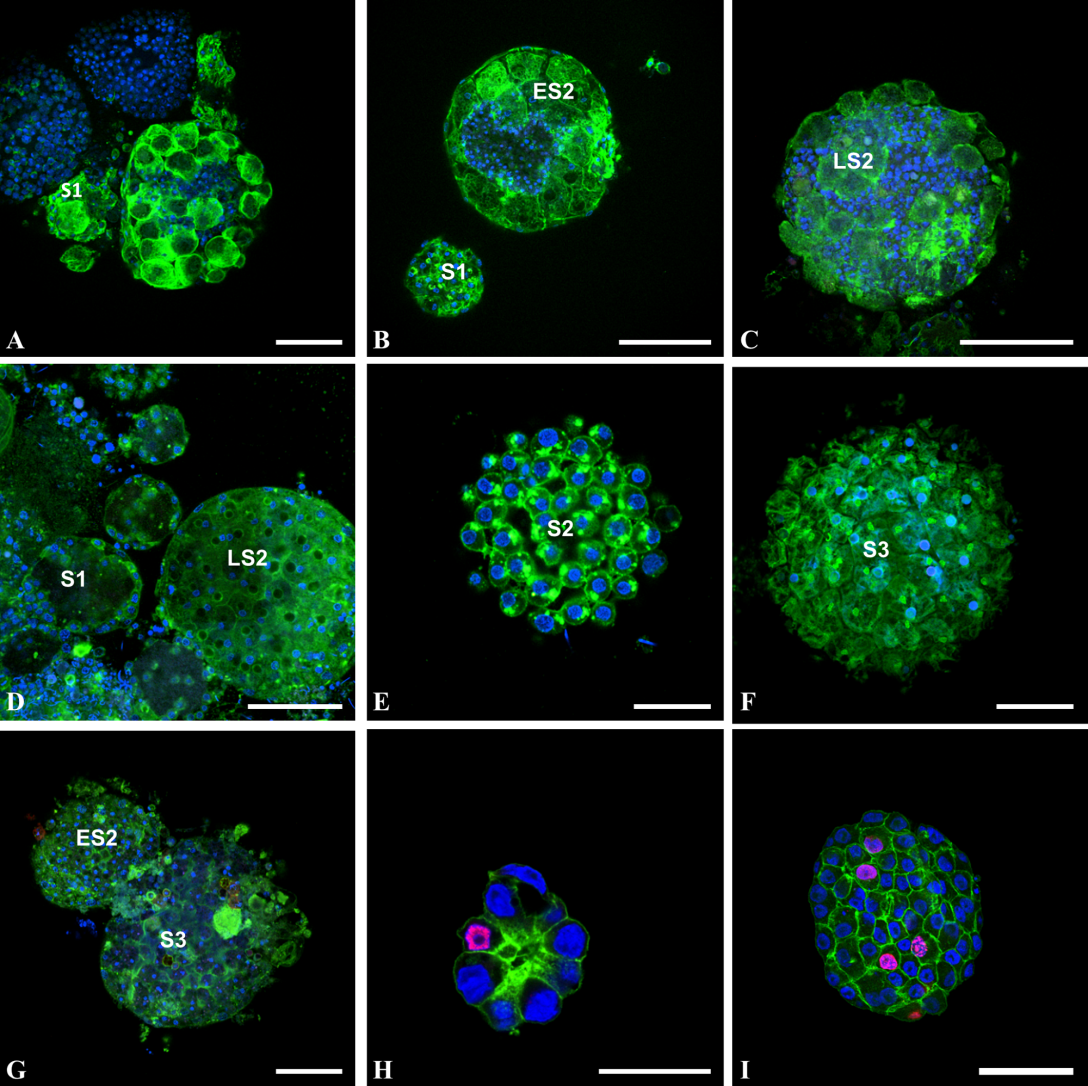
Cells do not proliferate during follicular morphogenesis of *Ciona intestinalis* (A-C), *Molgula citrina* (D-F), and *Phallusia mammillata* (G). Oocytes at different stages of differentiation (S1: stage 1 oocyte; ES2: early stage 2 oocyte; LS2: late stage 2 oocyte; S3: stage 3 oocyte) were collected and incubated overnight with 50μM BrdU, then fixed and stained with FITC-phalloidin (green pseudo-color), DAPI (blue pseudo-color) and anti-BrdU antibody (red pseudo-color). Note that in these oocytes, the follicular system is not fully achieved (as shown by phalloidin staining) and that no follicular cell was found BrdU positive. Note also that in areas devoid of follicular cells (A-C) blue DAPI-positive staining is indicative of the presence of numerous test cells. (H-I): confocal images of spheroid COS cells at two growing stages triple labeled with FITC-phalloïdin (green pseudo-color), TRITC-secondary antibodies directed against anti-BrdU antibodies (red pseudo-color) and DAPI (blue pseudo-color). BrdU positive cells appear in magenta pseudo-color and served as positive control for BrdU incorporation experiments. Bars (from A to I respectively) = 20, 30, 40, 30, 25, 40, 50, 40 and 60μm.

**Fig 2:**
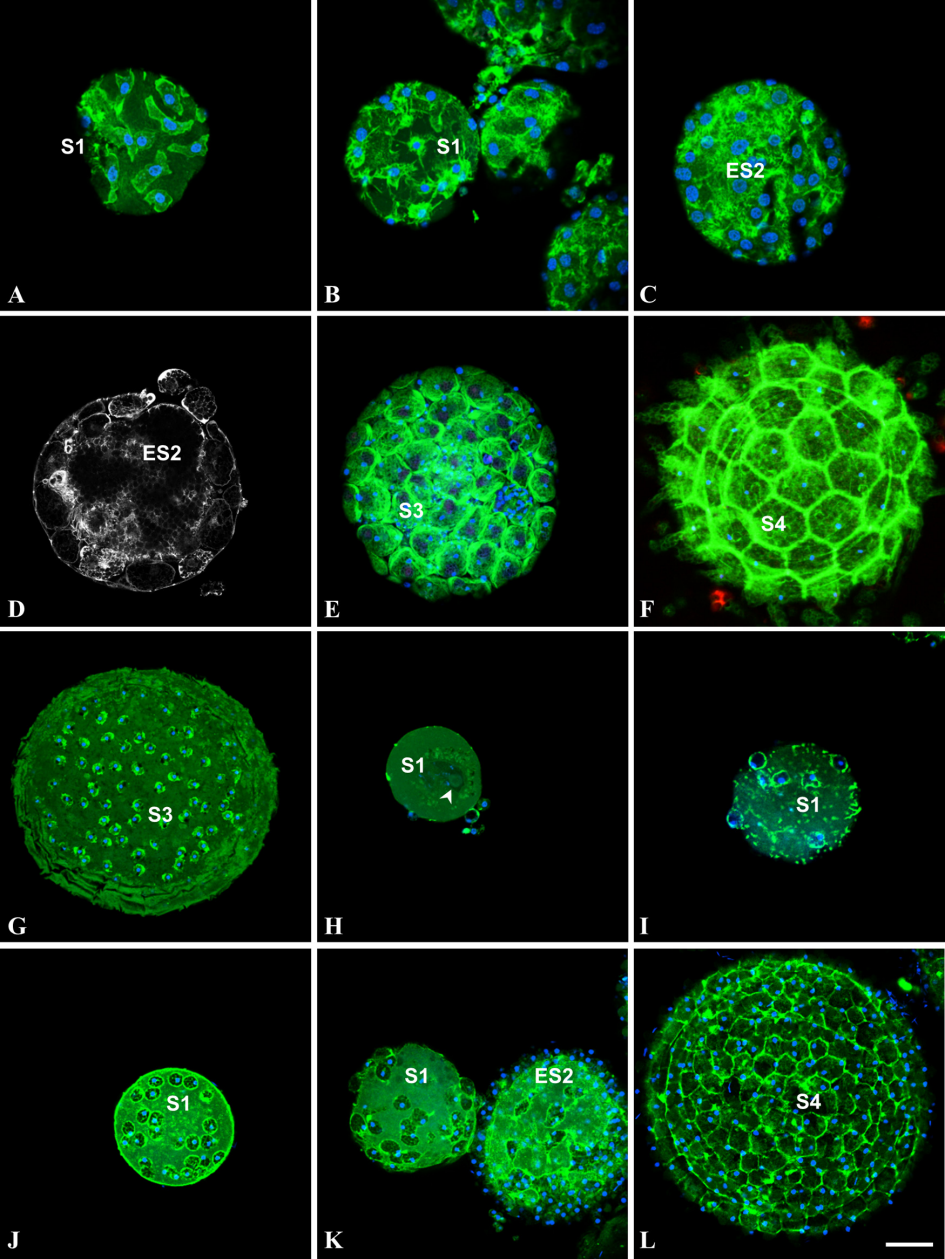
Cells do not undergo apoptosis during follicular morphogenesis of *Ciona intestinalis* (A-F), *Ascidiella aspersa* (G), *Phallusia mammillata* H,I) and *Styela clava* (J-L). (A,B,C,E,F): confocal images of oocytes, triple labeled with FITC-phalloïdin (green pseudo-color), TRITC-TUNEL (red pseudocolor) and DAPI (blue pseudo-color) after dislocation of *Ciona intestinalis* gonads. (D): actin staining with FITC-phalloïdin of another plane at the same stage of C in order to better visualize the accretion process. Note the elongated, fibroblast-like shape of follicular cells spread onto young oocytes (A). (G): Accretion process just before the terminal stage of *Ascidiella aspersa* oocyte maturation. (H, I): first stages of accretion process in *Phallusia mammillata*. (J-L): Accretion process in *Styela clava* from first stage to mature oocyte. S1: stage 1 oocyte; ES2: early stage 2 oocyte; S3: stage 3 oocyte; S4: stage 4 mature oocyte; arrowhead: germinal vesicle. Note that in areas devoid of follicular cells (E, K) blue DAPI-positive staining is indicative of the presence of test cells. Bar (from A to L respectively) = 5, 10, 15, 15, 20, 25, 25, 16, 16, 25, 25 and 22μm. Note that no apoptotic cell was detected in A-L, except the artifactual refringent bodies in F.

**Fig 3:**
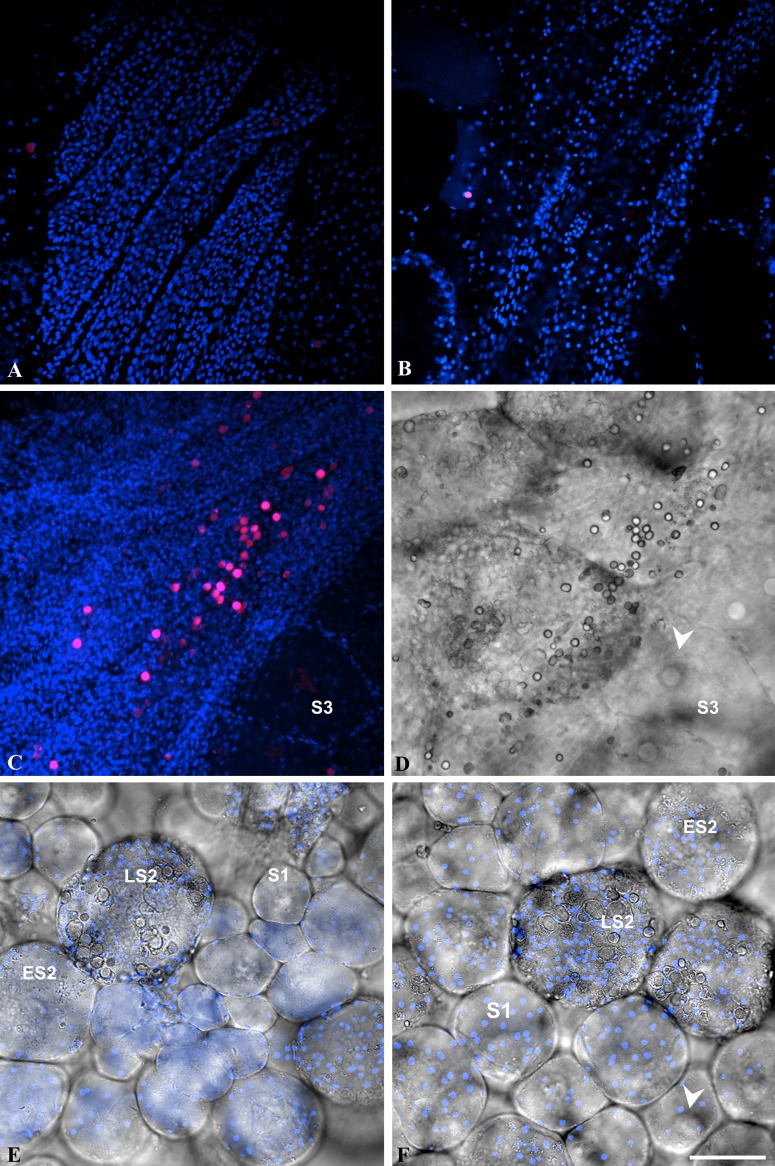
Localization of EdU positive cells in the gonad of *Ciona intestinalis*. A-B: double fluorescence of single confocal sections (bleu pseudocolor: DAPI staining; red pseudo-color: EdU labeling). C-D: double fluorescence, as in A-B, of stacked confocal sections (C) and (D) phase contrast image of the same field. E-F: mixed double fluorescence of single confocal sections, as in A-B, and phase contrast images of oocytes at various stages of maturation devoid of EdU labeling. Note that clustered EdU positive cells (magenta pseudo-color resulting from DAPI and EDU merged staining) were only detected at proximity of the gonad wall (C-D) but never in contact with oocytes whatever their stage of maturation. S1: stage 1 oocyte; ES2: early stage 2 oocyte; LS2: late stage 2 oocyte; S3: stage 3 oocyte; arrowhead: germinal vesicle. Bar = 50 μm.

Ascidians were collected in Roscoff (Bretagne Nord, France, latitude: 48.726199, longitude: -3.985324999999989) and their oocytes maintained at 18°C in 0.2 μm-filtered seawater containing 100U/ml penicillin, and 100 μg/ml streptomycin. Oogenesis occurs continuously throughout adulthood [13,14] and, oocytes, at different stages of folliculogenesis [13,15], were obtained through gonad dislocation (Figs 1 and 2) or observed into the whole organ after a single cut of the gonad wall (Fig 3). Experiments conducted on ascidians (marine invertebrates) did not require specific permissions and were collected outside of private or protected area. Our field studies did not involve endangered or protected species.

### Cell cultures

COS cells (COS-1 (ATCC CRL-1650), gift from the “Institut de Biothérapie”, Montpellier, France) were seeded either on standard Petri dishes (flat monolayers), or, to induce spherical monolayers, on hydrophobic Petri dishes in MEM alpha medium supplemented with 10% fetal bovine serum, 20ng/ml HGF, 10ng/ml EGF, 25mM glucose, 1μM thyrotropin-releasing hormone, 1μM hydrocortisone, 10μg/ml insulin, 50μg/ml albumin-linoleic acid, 0.1μM selenium acetate, 0.5μg/ml ferrous sulfate, 0.75μg/ml zinc sulfate, 10mM nicotinamide,100 μg/ml streptomycin and 100U/ml penicillin.

### Fly strains crosses for dIAP and PH mutants

Flies were grown on standard medium. GFP-labeled cells specifically blocking apoptosis in the developing wing blade were generated by crossing the following strains: *nub*-GAL4; UAS-GFP and UAS-DIAP1 kindly provided by the Bloomington fly stock Center. To induce hyperproliferation in eye discs through PH invalidation, we used the *ph* ^*505*^ allele^44^, which carries mutations in both the *ph-d* and *ph-p* genes forming the *ph* locus and the *Scm* ^*D1*^ null allele, as in [16].

### BrdU and EdU incorporation

The ascidian gonads and the COS cells were incubated overnight at 18°C (Gonads) or at 37°C (COS cells) in their respective medium containing 50 μM bromodeoxyuridine (BrdU) (sigma B5002, St Louis, MO, USA) and fixed with 1% paraformaldehyde overnight at 4°C. After 1h DNase treatment at 37°C, Gonads and COS cells were incubated with monoclonal anti-BrdU (GE Healthcare RPN 202) for 2h at 37°C. Appropriate secondary antibody was FITC-conjugated donkey-anti-mouse IgG (Jackson Laboratories). The Click-IT EdU imaging kit from Invitrogen was used as an alternative to BrdU labeling. EdU (5-ethynyl-2’-deoxyuridine) is a nucleoside analog of thymidine and is incorporated into DNA during active DNA synthesis [17]. Detection is based on a click copper-catalyzed covalent reaction between an azide and an alkyne. After dissection, gonads were opened and incubated overnight at 18°C with 50 μM EdU followed by paraformaldehyde fixation, permeabilization and DAPI staining.

### Labeling and fluorescence microscopy

Oocytes, COS cells and *Drosophila* wing and eye discs, were fixed and processed for cell contour (WGA or FITC phalloïdin labeling), DAPI and TUNEL staining (Roche, *in situ* cell death fluorescein or rhodamine detection kit) as previously described [10,18]. Specimens were analyzed with a Leica SPE laser confocal microscope (Montpellier RIO Imaging platform, France).

### Cell shape counting

The polygonal distribution of cells was manually determined from micrographs. Comparison with the similarly determined distributions for the same epithelium (wing disk) in Gibson et al.[12], as reported in the main Text, show that this procedure is repeatable. For spherical epithelia, a centered circle representing 80% of the surface of the half-sphere was drawn, and only cells within this circle were counted, as shapes of cells at the periphery of the half-sphere were impossible to determine as accurately.

### Statistical analysis

Statistical analyses were performed first to compare distributions of cell shapes among different conditions (*i*.*e*. different species or strains), taking into account that the shapes of different cells within an individual are not independent from each other by a mixed model with a random effect for individuals, nested within each condition. Cell shape frequencies were therefore fitted by a multinomial Generalized Linear Mixed Model (GLMM). The multinomial response is reduced to a trinomial response to avoid biases in the tests that could result from comparing small cell counts. This trinomial response can be fitted by two nested binomial GLMMs: in the first one, the response variable is the frequency of the most common shape (hexagons) against all other shapes; in the second one, the response is the frequency of the second most common shape against all shapes except hexagons. Such analyses are called joint binomial GLMMs. Different models were compared by likelihood ratio (LR) chi-square statistics. An LR test comparing cell shape frequencies in *n* conditions combines the independent LR statistics from the two nested binomial GLMMs each with *n*-1 degrees of freedom (df), and thus has 2(*n*-1) df. All analyses were performed using the R software (R Development Core Team, 2014) and the spaMM package [19] was used for fitting multinomial GLMMs.

### Numerical modeling

The numerical modeling takes into account the capacity for each cell to move in association with cell shape “plasticity” and, therefore, allows cells to self-organize. The model is based on the physics of divided media with contact and at-distance interactions [20,21]. It is built on sets of solid spherical units (here referred to as grains) undergoing interactions within the cell area (see Table 1 for a detailed list of the parameters used to run the simulations). For each cell, two sets of grains were considered. The first set, internal grains in contact, represents the cellular cytoplasm and hence is characterized with rigidity in compression but not in traction. The second, peripheral grains, represents the cell plasma membrane connected by tensile “cable” elements. The size of grains was a balance between cell resolution and computational time (the smaller the grains, the better the epithelial tightness, and the longer the processing). For a defined area (imposed by a finite number of grains), a cell can be deformed within a large variety of polygonal cell shapes. The model also took into account the existence of cell-cell contacts (*i*.*e*. cadherins), and implied that once two cells interact, they do not dissociate afterwards. In the case of accretion, small native cells successively contact the spherical support with a random positioning. Then, they undergo a step of cell growth until the maximal size (experimentally determined) is achieved. During this period of time, the cells have the capacity to move freely on the surface and to develop contact interactions with neighboring cells that result in shape modifications.

**Table 1:** List of parameters used to run computer simulations of epithelial morphogenesis.

Geometry (1)	
- ***grain***: size: five different diameters	Radius of grain (2): 2.5; 2.8; 3.0; 3.3; 3.5 μm
number in a cell: variation depends on scenario	- accretion: 3 to 15 grains at the beginning
	(the later the cell arrives, the bigger it is)
	- proliferation: 6 grains at the beginning
	- accretion and proliferation: 41 grains at the end (mature cell)
- ***cable***: size	diameter 3.0 μm
length: variation between Lmin and Lmax	Lmin = 2 μm; Lmax = 5.5 μm
number: variation depends on length	cable removed if length < Lmin; split in two if > Lmax
- ***cell/cell adhesion***: “cadherin” like junction	possibly created if cable distances of two cells < 13 μm
	(only half of candidates are really created)
- ***cell division***: contractile actin filament	passes through the mature cell barycentre
	connects membrane opposite parts (shortest path)
**Mechanics**	
- ***grain and cable***: density	0.9
- ***grain/grain contact***: compressive stiffness and damping	Egg = 20 N/m; Cgg = 5 N/(m.s^-1^)
- ***grain/cable contact***: compressive stiffness and damping	Egc = 13 N/m
- ***cable***: tensile stiffness	Ec = 5.10^–3^ N/m
tension: variation between Tmin and Tmax	Tmin = 0.05 N; Tmax = 0.30 N
**Biology** (3)	
- ***organization rate***	number of time steps between two consecutive stages
- ***cell growth***	1 grain added
	(its diameter depends on the number of existing grains)
- ***tissue growth***: depends on scenario	- accretion: 2 new cells maximum arrive
	(if there is enough space after testing 100 random positions)
	- proliferation: mature cells undergo mitosis or apoptosis
	(considering 7% of cells in mitosis and a variable rate of apoptosis/mitosis equal to 1/3, 1/5 or 0)
**Numerical**	
- ***time step***: used in 4^th^ order Runge-Kutta scheme	0.004 s (see equation in materials and methods)

^(1)^ “grain” refers to a cytoplasmic grain; “cable” to a membrane tensile cable element.

^(2)^ Sequential arrival order of grain inside the cell: 2.5; 2.8; 3.0; 3.3; 3.5; 2.5; 2.8…

^(3)^ A “stage” corresponds to the time when these events occur: cells grow (grains are added and cables are modified), new cells arrive (in accretion scenario) or cells divide/die (in proliferation scenario).

In proliferation mode, several small new cells are randomly generated on the spherical support. Then, they grow and after reaching the maximal allowed size, some cells are randomly “selected” to divide (mitosis) or to die (apoptosis). When a division occurs, the cell is split into two identical daughters that then evolve independently. When apoptosis occurs, all grains of the cell are gradually removed. From a mechanical point of view, the system evolves according to the equations of solid dynamics. Mechanical equations were solved using an explicit time integration approach that first determines the particle accelerations in the actual state according to the contact forces exerted between grains, and then their velocities and positions. This choice is governed by the highly non-linear nature of the simulated system. The time-history progress is therefore computed in a 4^th^ order Runge-Kutta scheme (RK4) based on the evaluation of the function, the first derivative of the dynamical state *X* of the system which is

$$f(X,t)=\frac{d\;X(t)}{dt}=\sum_{p}\left\{\begin{array}{c} {\dot{x}}_{p}(t)\\ {\ddot{x}}_{p}(t) \end{array}\right\}=\sum_{p}\left\{\begin{array}{c} {\dot{x}}_{p}(t)\\ F_{p}/m_{p} \end{array}\right\}$$

Where *x*_*p*_ *(t)*, ${\dot{x}}_{p}(t)$ and ${\ddot{x}}_{p}(t)$ are, respectively, a particle *p* position, velocity and acceleration at time *t* and *F*_*p*_ is the sum of the force applied to this particle.

Cytoplasmic forces depend on the unilateral grain/grain or grain/membrane contact (with a linear elasticity in compression). Peripheral membrane forces are based on the cable element stiffness (linear elasticity in traction). However, to avoid excessively high forces consecutive to the cell growth, the unrestrained length of these elements increases in parallel to the number of internal grains, resulting in a quasi-constant tension. External forces depend on membrane/membrane interactions when cells are in contact.

Grain mass is prescribed according to the cell average density (0.9 g/mL). A small viscosity was introduced (Kelvin-Voigt model: viscous damper and elastic spring connected in parallel) to control convergence of iterative calculations and slow down oscillations close to the equilibrium positions. The positioning onto the support was ensured via geometrical constraints (constant distance of every grain to the center of the spherical support). The counting of neighboring cells was numerically performed by checking membrane/membrane contacts.

## Results and Discussion

### Ascidian folliculogenesis results from an accretion process leading to highly ordered epithelial topology distinct from proliferative models

We showed previously that the pattern of *Ciona intestinalis* external follicular cells exhibit a regular geometric packing [11]. We extended this characterization to four other ascidian species and asked how this spherical epithelial follicular system is formed during oocyte maturation and growth. Using BrdU incorporation we first tested whether follicular cells proliferate when anchored to the unfertilized eggs. Ascidian gonads were incubated overnight with BrdU, then oocytes were released from gonads, fixed, permeabilized and stained with anti-BrdU antibody (Fig 1). Remarkably in no species (Fig 1: *Ciona intestinalis* A-C; *Molgula citrina* D-F; *Phallusia mammillata* G), whatever the stage from S1 to S3 [15] of oocyte maturation was, no signature of BrdU incorporation was recorded in accessory, follicular and test cells. In the same experiment the BrdU-positive COS cell spheroids provided a positive control (Fig 1H and 1I). Further analysis of the ascidian oocytes revealed that younger immature oocytes were incompletely covered by follicular cells suggesting that the completion of morphogenesis at the oocyte surface did not involve mitotic events. Reciprocally we also checked for the potential presence of apoptotic events using TUNEL labeling **(** Fig 2). Again whatever the ascidian species or the maturation stage from S1 to S4 [15] (Fig 2: *Ciona intestinalis* A-F, *Ascidiella aspersa* G, *Phallusia mammillata* H-I and *Styela clava* J-L) no detection of apoptosis was recorded in any type of accessory cells. It is of note that on immature *Ciona intestinalis* oocytes test cells were observed in surface areas devoid of external follicular cells (Fig 1B and 1C; Fig 2E), suggesting that these cells covered the oocyte prior follicular cells. The size of follicular cells appeared to grow throughout the various maturation stages from 5 μm length in S1 **(** Fig 2A **)** to 30 μm in S4 **(** Fig 2F **)**. In younger stages follicular cells adopted an elongated, fibroblast-like shape **(** Fig 2A **)** suggesting that these cells at this stage possess spreading and migrating properties. Additional ultrastructural studies would be required to characterize whether follicular cells are spread onto a chorion matrix already present at this stage of maturation.

These two sets of results argued for a hypothetic mechanism of epithelial follicular morphogenesis which would be described by a sequential order of events including a proliferation step away from the oocyte surface and a migration step towards the oocyte. Once anchored to the egg, cells would potentially grow and assemble to each other to complete the covering of the oocyte surface as a highly organized epithelial monolayer. Folliculogenesis would therefore be the result of successive additions of cells onto the oocyte surface through a cumulative process referred to as accretion. Where these follicular cells would come from? To try answering the question we performed EdU incorporation into the whole gonads of *Ciona intestinalis*, fixed the gonads and analyzed where proliferative cells could be detected in the entire organ **(** Fig 3 **)**. In agreement with the BrdU incorporation reported above, no cell associated with oocyte was found labeled, whatever its maturation stage from stage 1 to 3 [15]. As shown in Fig 3A and 3B, large areas of the gonads were EdU-negative. Interestingly, clustered groups of EdU-positive cells, never associated with oocytes, were located close to the organ wall (Fig 3C and 3D **)**. These cells might represent a niche of follicular cell precursors as proposed by Cloney in 1995 [22]. However, the lack of a specific cell marker linking these proliferative cells to follicular cells prevent any definitive conclusion. Overall these results argue that the assembly of follicular cells on top of the oocyte surface takes place within the gonads through a cumulative accretion process. The most important point is that once follicular cells reached the oocyte surface, by migration from a still unidentified proliferative niche, no further mitotic or apoptotic events occur. Folliculogenesis in ascidians can therefore be considered as an example of non-proliferative epithelial morphogenesis. Additionally, since a similar mode of morphogenesis by accretion was documented in the five studied species this morphogenetic process might be widespread among solitary ascidians.

**Fig 4:**
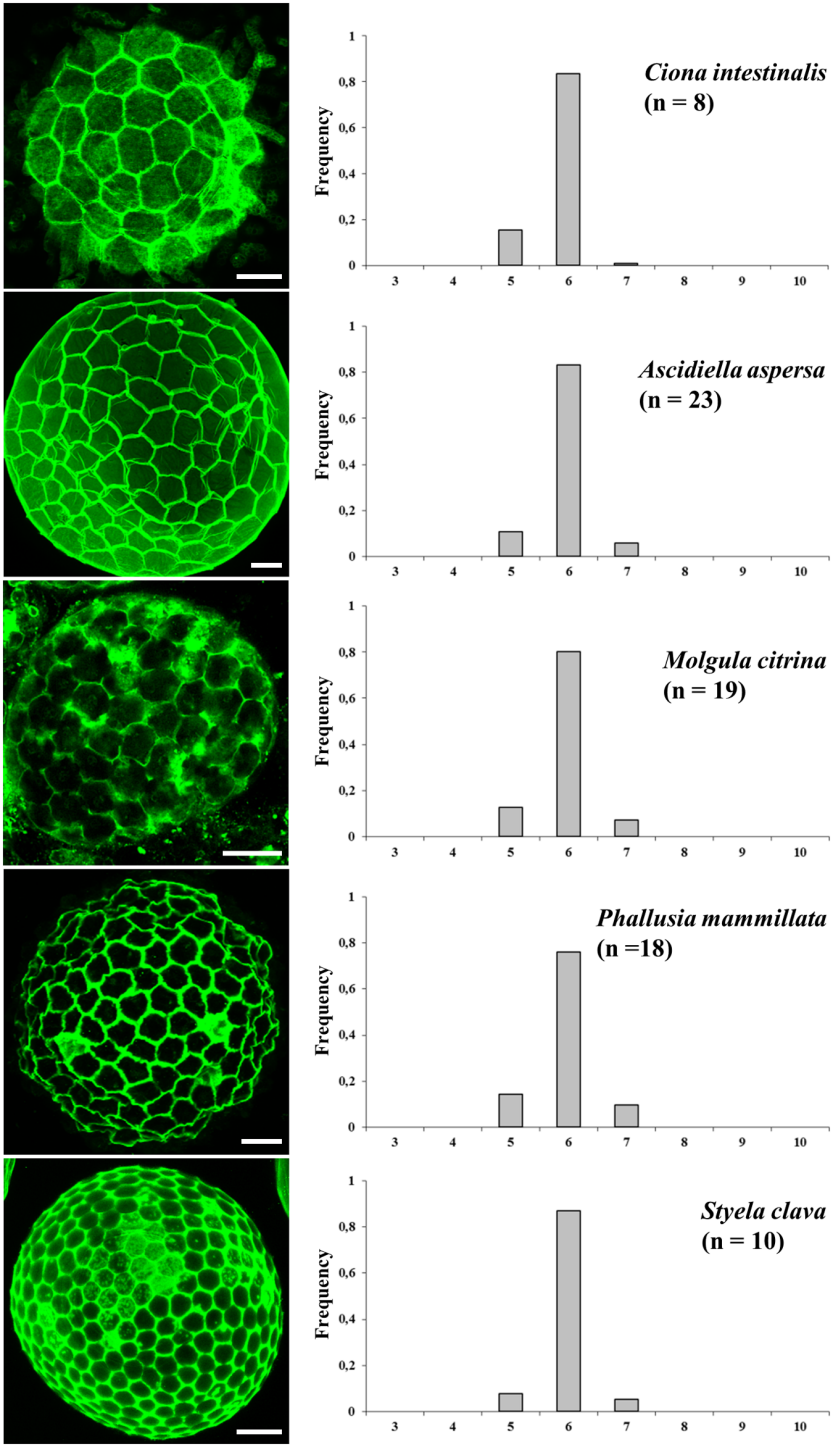
Topological organization of non-proliferative epithelial follicular cells in ascidians. Cell membranes were labeled with FITC-WGA in fixed oocytes (left panels). The percentage of cell shapes (the shape of one cell being directly deduced from the number of its neighboring cells as indicated in the abscissa of histograms) was manually determined for each indicated species. All epithelia are exclusively organized with hexagons, pentagons and heptagons. n: number of experiments. All standard errors were lower than 0.09. Bars = 35μm.

We then characterized further the topological organization of follicular cells in the five ascidian species and quantified the polygonal distribution referred to as cell shape, *i*.*e*. the number of neighboring cells (Fig 4). A restricted distribution frequency of pentagons, hexagons and heptagons characterized *Ciona intestinalis* follicular topology. Interestingly a similar topology is maintained throughout species with widely different numbers of follicular cells (*i*.*e*. 60 in *Ciona intestinalis*, 150 in *Ascidiella aspersa*, 200 in *Molgula citrina*, 300 in *Phallusia mammillata* and 350 in *Styela clava*) with significant (joint binomial GLMMs, LR chi-square = 32.8, df = 8, p = 6.5x10^-5^; see Methods for details of the test) but small variation among species around frequencies of ~80% hexagonal cells and the remaining 20% shared by pentagonal and heptagonal cells. Remarkably, within the limits of this analysis, the topological features remained invariant whatever the value of the surface occupied by a single cell. For comparison the covered area by one *Ciona intestinalis* follicular cell was found 4.7 times, on average, larger than that by one *Styela* cell.

**Fig 5:**
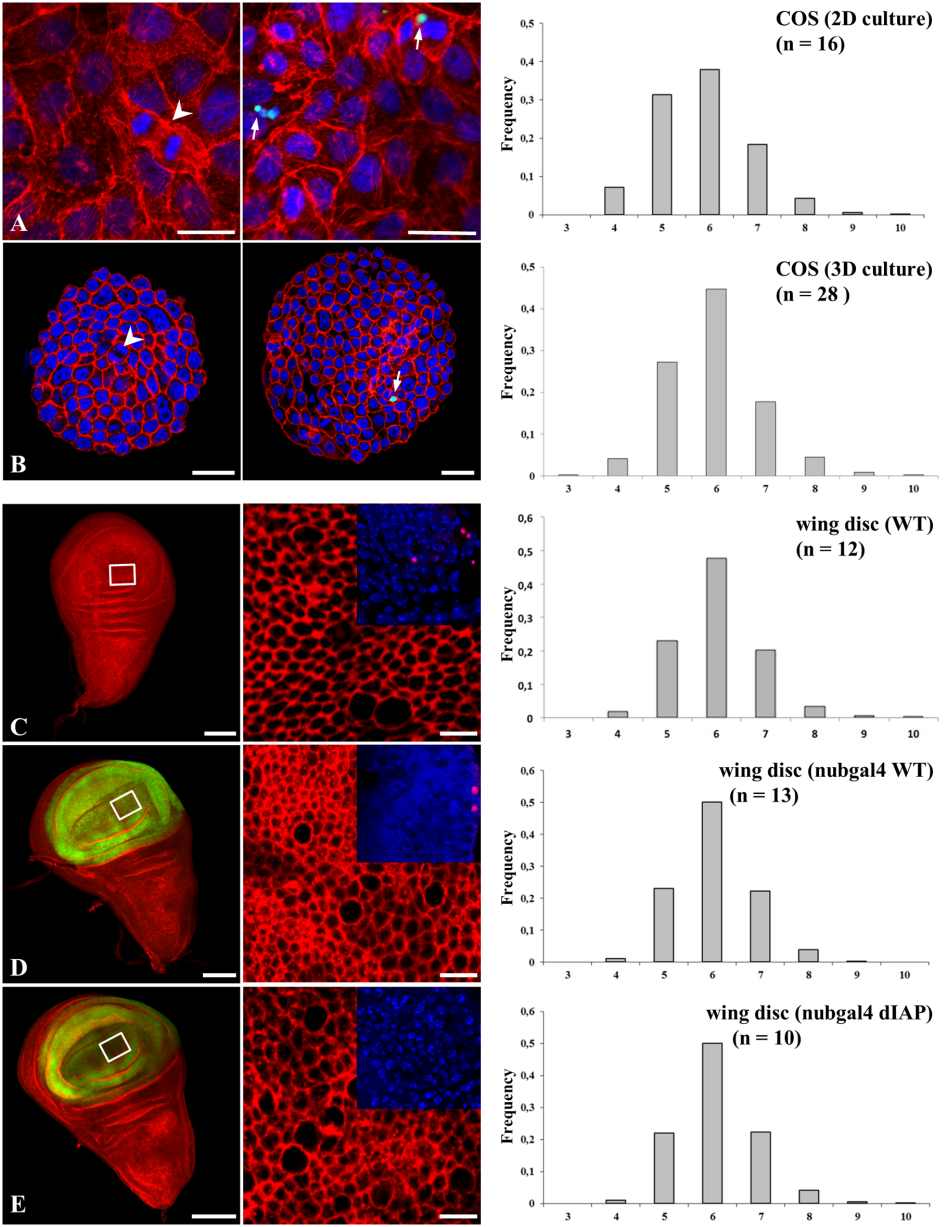
Topological organization is significantly modified in proliferative epithelia. The polygonal cell frequency was determined, as in Fig 4, for flat (A) or spherical (B) COS cell monolayers and *Drosophila melanogaster* wing discs (C-E). Unless otherwise indicated, actin is color-coded red, nuclei blue. Arrows point to TUNEL-positive COS cells (green), and arrowheads to mitotic cells. Wing discs, wild type (C), expressed green-coded GFP-nubgal4 empty driver (D) or GFP-nubgal4-dIAP (E). In inserts (C-E), TUNEL-positive cells are color-coded red. n: number of experiments. All standard errors were lower than 0.07. Bars = 10μm (except C, D, E left panels: 100μm).

Since follicular cells from *Ciona intestinalis* and other ascidians represent models of non-proliferative epithelia topological organization we aimed to compare the distribution frequency with that of proliferative epithelia. The choice of ascidian follicular epithelium implied the study of a spherical epithelial monolayer raising the question as to whether direct comparisons can be performed between spherical and flat epithelia. We, therefore, first studied COS epitheloid simian cells as a convenient alternative to oocytes as these cells are proliferative as evidenced by BrdU incorporation (Fig 1H and 1I) and can form either flat or spherical monolayers **(** S1 Fig). We found that COS cell monolayers were identically organized either being cultured as spheroids or as flat monolayers (Fig 5A and 5B respectively). COS cells displayed a much lower percentage of hexagons (~45% compared to 80% in ascidians), and higher frequencies of pentagons and heptagons with the occurrence of a significant number of cells with 4, or 8 to 10 neighbors. We next turned to the *Drosophila melanogaster* imaginal wing disc model as an example of proliferative flat epithelium, even though it might as well be considered as a finite invaginated epithelial sac. This model, because it is susceptible to genetic manipulation [23], has been extensively used to understand how geometric order emerges [12]. Wild type wing discs (Fig 5C) were characterized by a level of distribution frequency of cell shapes similar to what was previously published (with hexagon frequency = 0.49 and pentagon frequency = 0.24 in our data, vs. 0.46 and 0.28, respectively, in the published data [12]) and with the same characteristics described above in the COS cell model. Therefore we concluded that there exists a clear partition in the frequency of topological distribution between ascidian follicular epithelium and models of proliferative epithelia such as cultures of COS cells and *Drosophila melanogaster* wing discs.

**Fig 6:**
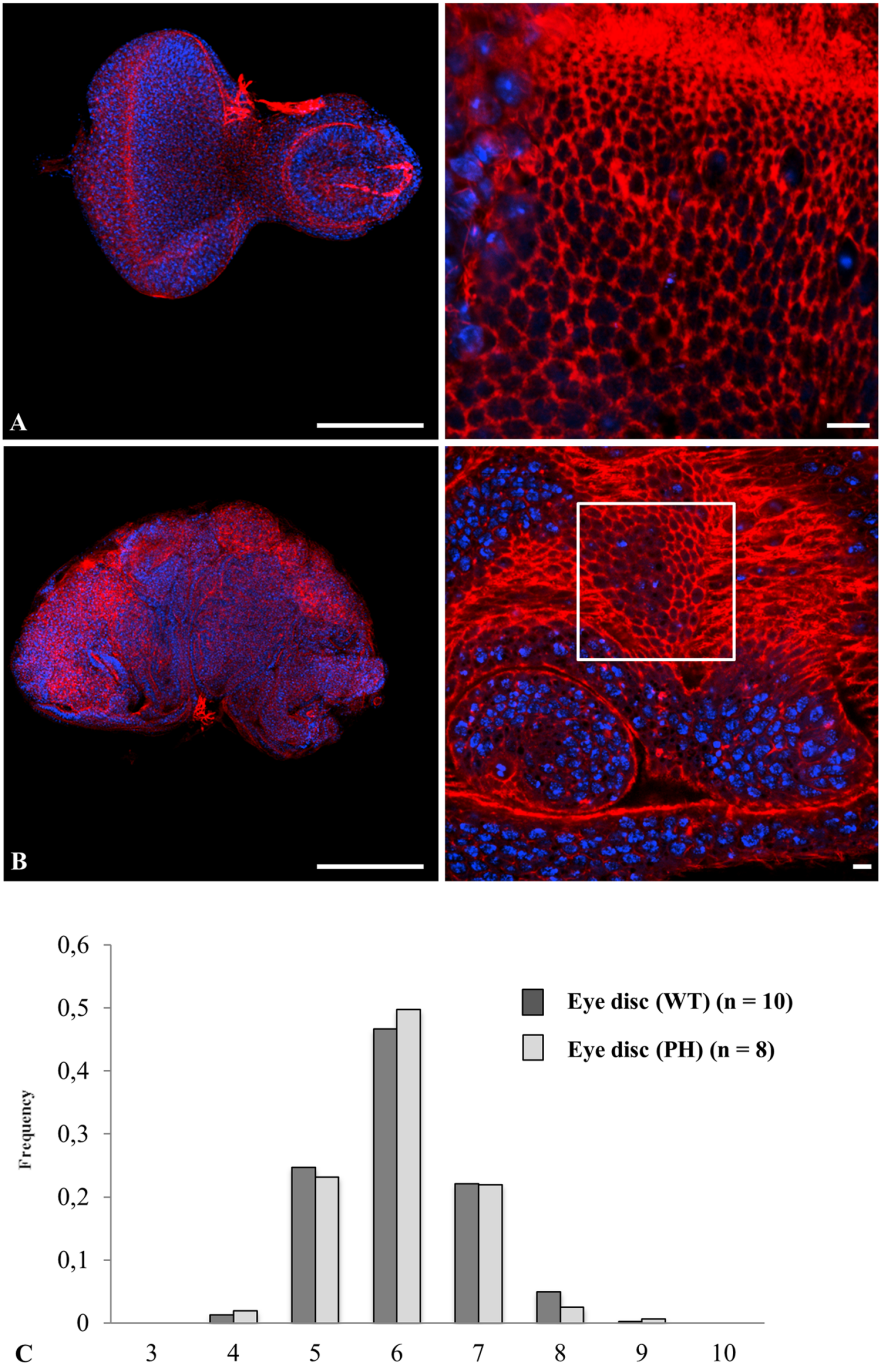
Hyper-proliferation does not impact the topological features of proliferative epithelia. A (wild type) and B (PH mutant) of *Drosophila* eye discs double labeled for actin (red pseudo-color) and nucleus (blue pseudo-color). The polygonal cell frequency (C) was determined, as in Fig 4, for wild type (dark grey) or PH mutant (light grey). n: number of experiments. All standard errors were lower than 0.08. Bars = 100 μm (left panel) and 10 μm (right panels).

Since follicular cells from *Ciona intestinalis* and other ascidian species could be considered as a non-proliferative epithelium, it is worth considering the extent of divergence and/or convergence that define the topology between non proliferative and proliferative epithelia. Proliferative cells and tissues are known to be subjected to apoptosis and this was identified as a dynamic force required during dorsal epidermis closure [24] or epithelium folding [25]. Both COS and *Drosophila melanogaster* wing disc cells exhibited a low level of apoptosis affecting ~ 2.0% of wing disc cells at any time on average (Fig 5A and 5B right panels, inserts C-D and [26]). In order to discriminate how proliferative or apoptotic events impact on topology, we next invalidated apoptosis in the wing disc model. Over-expression of the *Drosophila melanogaster* inhibitor of apoptosis protein (dIAP) completely abrogated apoptosis in the *Drosophila melanogaster* mutant context (Fig 5 inserts E). Wing discs that over-expressed dIAP (Fig 5) exhibited a similar distribution frequency of the different cell shapes as compared with that found in the wild type and the control nub gal4-treated epithelia (compare Fig 5E with Fig 5C and 5D). The frequencies of pentagons and heptagons differed by 1.5–2% between control and treatment, which is nevertheless statistically significant (joint binomial GLMM, LR chi-square = 12.06, 2 df, p = 0.0024). We then induced hyperproliferation in *Drosophila melanogaster* eye disc in order to try to test the effect of a hyperproliferative state. However hyperproliferation, as promoted, resulted in tumor induction **(** Fig 6B **)** with epithelial cells organized as a three-dimensional tissue of multilayered epithelium [16] from which topological organization cannot be directly compared with wild type monolayered epithelia **(** Fig 6A **)**. We therefore focused to quantify the topological organization of hyperproliferative monolayered cells adjacent to multi-layered tumors (window in Fig 6B right). The topological organization of these cells was found unchanged from that of wild type animals **(** Fig 6C **)** (joint binomial GLMM, LR chi-square = 0.42, df = 2, p = 0.81), supporting the conclusion that the tissue is robust enough to resist mechanical forces exerted by neighboring proliferative tumors. Our data suggest that tumor proliferation generating multilayered organization did not change the frequency of cell shapes that can be determined in areas of the disc where the monolayer organization was conserved. Overall, a wide variation in cell shape frequencies was detected among all seven proliferative conditions (*i*.*e*. flat and spherical COS cells, Wild type, nub-GAL4; UAS-GFP, and UAS-DIAP1 wing discs, wild type and PH eye discs) (joint binomial GLMM, LR chi-square = 72.58, df = 12, p = 1x10^-10^). The results clearly indicate that although a slight but significant change in distribution frequency distinguishes epithelia lacking apoptosis or invalidated for PH from control, apoptosis or hyperproliferation *per se* does not exert any major contribution to topological organization. Therefore our data clearly point that what distinguishes ascidians follicular epithelia from COS cells or imaginal discs cells is the proliferative character of the latter models.

We have described the occurrence of a restricted variation in cell shape frequencies among a set of non-proliferative epithelia, as compared with the wider one existing in well-characterized proliferative ones, and indicative that a clear partition does exist in the morphogenesis of the two sets of epithelia. We had previously shown in *Ciona intestinalis* that follicular cells are organized in a regular order, including several symmetries, based on the peculiar specificity that *Ciona intestinalis* oocyte is covered with a nearly constant number of 60 follicular cells. In this study, it is striking that the distribution frequency of cell shapes is closely maintained in other ascidian species whatever the number of follicular cells is (up to 350). By contrast, this distribution is markedly different when proliferation events take place during morphogenesis, with a significant decrease in hexagons and correlative increase not only in pentagons and heptagons but also the appearance of 4 and 8- to 10-sided polygons. Proliferative and non-proliferative epithelia differ (joint binomial GLMM taking into account the heterogeneity within these two categories as a random effect, LR chi-square = 62.43, df = 2, p = 2.8x10^-14^, with much larger variation among these two categories than within. For hexagonal shapes, for example, the fitted frequencies (including random effect predictions) vary within 0.383–0.535 among proliferative epithelia, and within 0.767–0.863 among non-proliferative ones. The different types of distribution of cell shapes therefore mostly depend on the proliferative status of the epithelia. Indeed, even though we have characterized only a few experimental models we were able to rule out that either the geometrical constraints (spherical or plane) or the number of cells or the involvement of apoptosis events play any comparable role in the definition of the distribution of cell shapes. In the proliferative context the distribution is extended to ranges from 4 to 10, as compared to 5 to 7 in the non-proliferative context.

### Cell proliferation increases the topological heterogeneity in simulations of epithelial morphogenesis

In order to strengthen the above conclusions we next developed computer simulations of the various morphogenesis processes. We simulated the development of finite objects, and specifically of spheres that could be directly compared to the spherical structures observed in ascidian eggs. We developed two scenarios for the evolution of the model: accretion or proliferation, and, for proper comparison of both, we used in each case the digital parameters that fitted *Ciona intestinalis* folliculogenesis.

**Fig 7:**
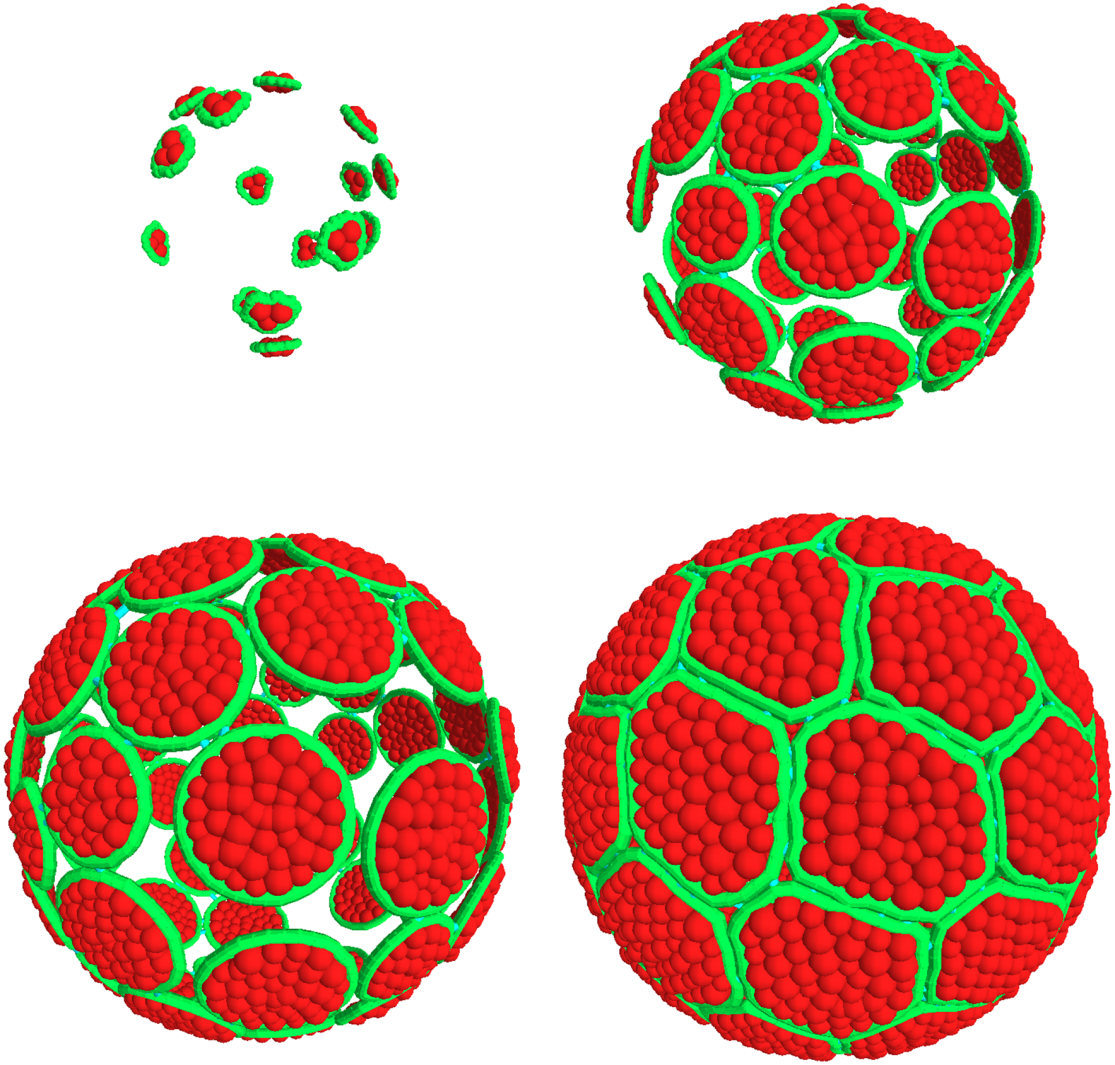
Simulation of the accretion scenario. Four successive steps of epithelial morphogenesis are represented with cell membrane contours in green and cytoplasmic grains in red; the process begins with 15 cells contacting the support, followed by cell growth and the successive addition of cells, up to a limit of 60 (as in *Ciona intestinalis*), that eventually assemble as hexagons and pentagons and entirely cover the sphere surface.

In the case of accretion (Fig 7), two driving parameters were found to be essential to the morphogenesis simulation: the “accretion rate” (the number of new cells added to the system in a given time) and the “cell growth rate” (the increase of the number of internal grains during the same period, see Methods for the definition of grains and Table 1 for diameter and number of the grains). These two parameters characterize the “organization rate”: low rates indicate that existing cells have more time to evolve and organize themselves before they grow again or that a new cell is added to the support (*i*.*e*. the organization rate increases). In proliferation mode, the driving parameters include a “cell division rate” and a “cell death rate” in addition to the cell growth rate already considered in the accretion model. Cell shrinking was mimicked by a drop in the number of grains.

**Fig 8:**
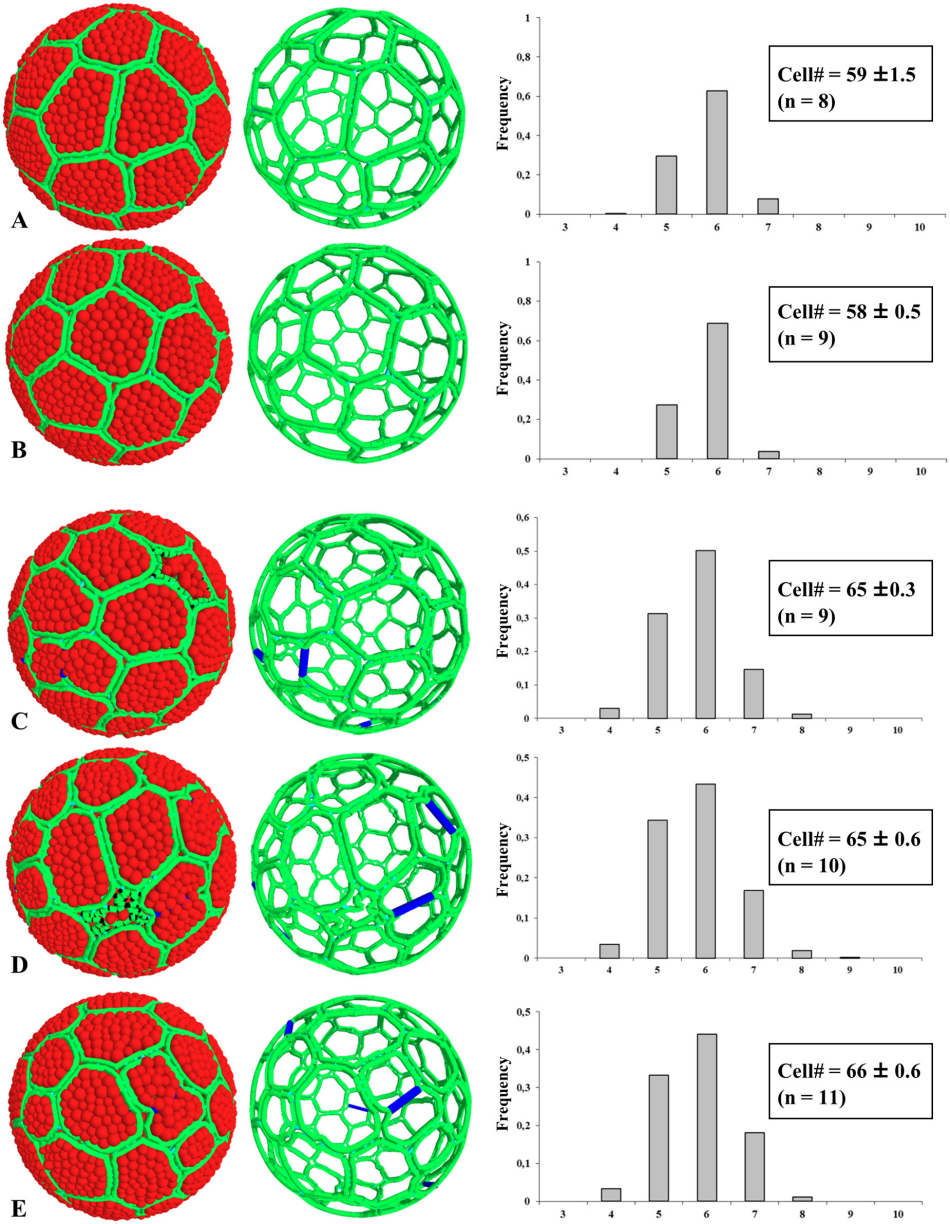
Simulations of epithelial morphogenesis in non-proliferative and proliferative context. Left pictures represent numerical objects at 99% coverage of the sphere surface (color-coded as in Fig 7, with blue lines indicating the axis of division). Apoptotic cells presented a twisted membrane and a loss of internal grains. Respective right skeleton pictures illustrate the 3D perspective. A-B: accretion-driven morphogenesis at two different organization rates (A, low rate; B, high rate, see also Table 1). C-E: morphogenesis in a proliferative context (7% mitotic cells at any time) with different mitosis/apoptosis ratios: 1/3 (C); 1/5 (D); no apoptosis (E). The number of cells covering the surface of the sphere is indicated in each case with the standard deviation from n number of simulations. All standard errors for histograms were lower than 0.09.

Model predictions in accretive and proliferative situations differed in two ways, accretion resulting in narrower distributions of cell neighbors (only pentagons, hexagons and heptagons present; Fig 8A and 8B). This distribution was strikingly similar to that determined experimentally for ascidians, and particularly with a close similarity in the growth of follicular cells and oocytes (Fig 7) mimicking experimental data observed in *Ciona intestinalis*. We then applied three simulations of morphogenesis under proliferative situations (Fig 8C–8E **)** using a fixed percentage (7%) of cells undergoing mitosis at any time and three different apoptosis/mitosis ratios: one apoptotic event for three mitotic events (Fig 8C, see also S1 Movie), one for five **(** Fig 6D **)**, and no apoptosis **(** Fig 6E **)**. For each simulation, designed with *Ciona intestinalis* cell parameters, the distribution frequency of cell shapes was found unchanged and perfectly matched the experimental data determined in proliferative epithelia, *i*.*e*. COS cells, *Drosophila melanogaster* wing and eye discs. From the various scenarios tested it could be concluded that, through the use of a model based on the physics of divided media, one can reproduce how proliferation impacts on epithelia morphogenesis and results in different distributions in cell shapes between non-proliferative and proliferative tissues.

In conclusion, we showed that follicular cells in ascidians are organized as a non-proliferative epithelium which develops through a process of accretion consisting of the sequential addition of a finite number of cells on a spherical template. This mode of morphogenesis complies with physical rules and leads to highly ordered tissue characterized by a very narrow range of cell shapes (5 to 7 neighbors). In contrast proliferative epithelia are less highly ordered (4 to 10 neighbors) and this loss of organization appears as an adaptive plasticity response to the constraints imposed solely by mitotic events. Indeed, some degree of topological heterogeneity would help for instance maintaining the impermeability function of epithelia all along its life time from embryogenesis to recurrent periods of cell renewal. Proliferation appears to be the major responsible mechanism to this increase of the topological heterogeneity, since the latter remains unaffected through the changes in the geometry of the tissue, the number of cells or by the invalidation of apoptosis.

## Supporting Information

S1 Fig COS cell spheroids grow as monolayers. Note the spherical monolayer organization of COS cells as evidenced by confocal microscopy centered sectioning. COS cells were double labeled for actin (red pseudo-color) and nucleus (blue pseudo-color). Bar = 15 μm.
(TIF)

S1 Movie Simulation of morphogenesis in proliferative epithelia. A 7% rate of mitosis with a 1/3 apoptosis/mitosis ratio was imposed at any time. Native cells were randomly positioned and evolved on the spherical support through individual growth, cell-cell (cadherin) interaction, mitosis and apoptosis and eventually resulted in the formation of an epithelial monolayer. The simulation was stopped when 99% of the surface was covered. The colour code is as in Figs 7 and 8.
(MOV)
