# Supplementary figures and images for: The Non-Proliferative Nature of Ascidian Folliculogenesis as a Model of Highly Ordered Cellular Topology Distinct from Proliferative Epithelia
Source: PLoS One. 2015 May 22;10(5):e0126341. doi: 10.1371/journal.pone.0126341 (PMC4441440; doi:10.1371/journal.pone.0126341)

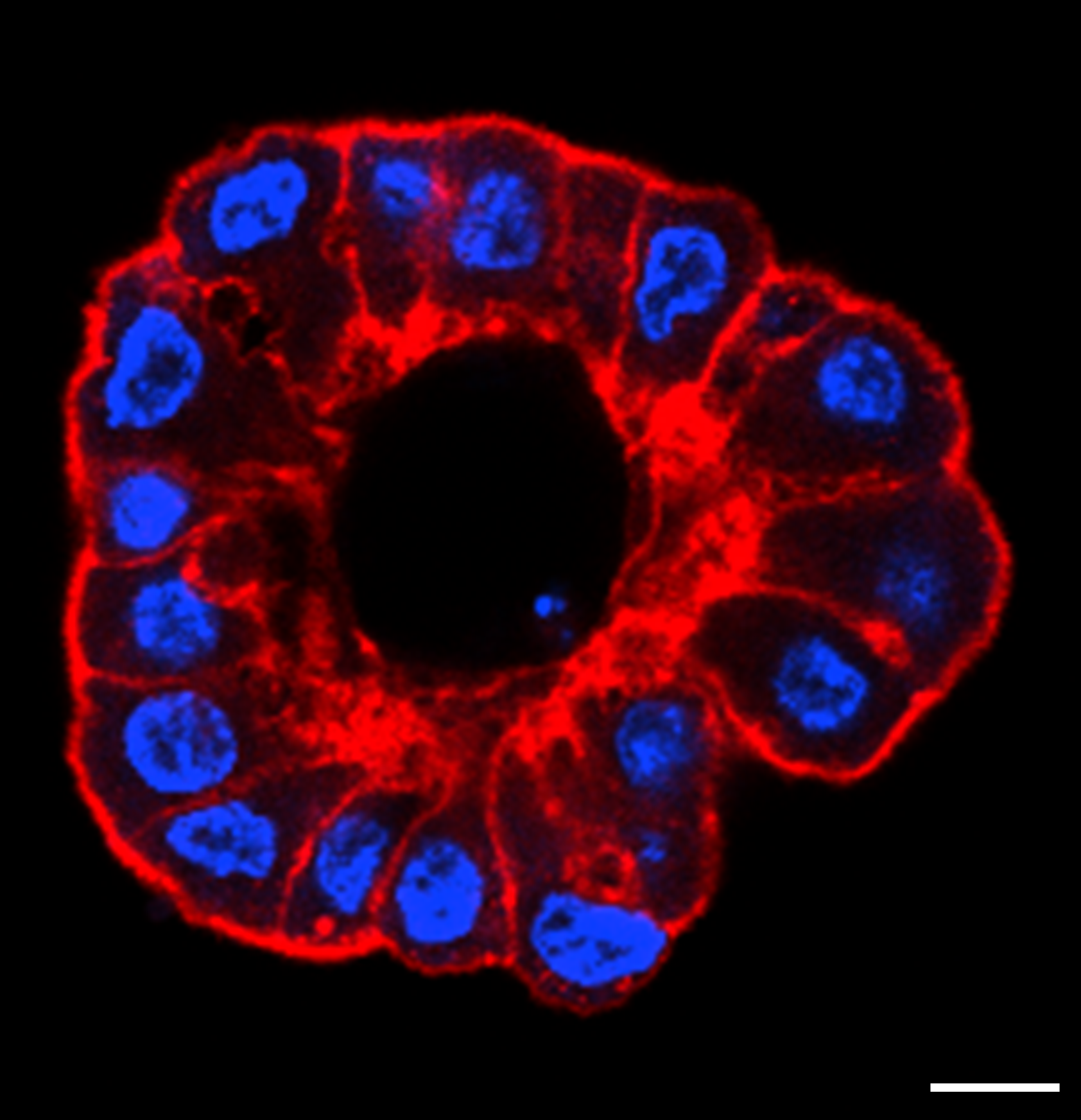

Supplement: S1 Fig — Note the spherical monolayer organization of COS cells as evidenced by confocal microscopy centered sectioning. COS cells were double labeled for actin (red pseudo-color) and nucleus (blue pseudo-color). Bar = 15 μm. (TIF) [file pone.0126341.s001.tif]
